# Psychometric evaluation of a psychosocial wellbeing questionnaire for older adults in Sibu, Sarawak, Malaysia

**DOI:** 10.1186/s12877-026-07257-5

**Published:** 2026-03-04

**Authors:** Jeffery Stephen, Lim Siong Hee, Sam Froze Jiee, Md Mizanur Rahman, Rosalia Saimon, Nur Azimah Azman, Gui Hun Chuen, Romano Ngui

**Affiliations:** 1 Department of Community Medicine, Faculty of Medicine and Health Sciences, Universiti Malaysia Sarawak, Datuk Mohammad Musa Road, Kota Samarahan, 94300 Sarawak Malaysia; 2 Sarawak State Health Department, Samarahan Divisional Health Office, Samarahan, Sarawak Malaysia; 3 Department of Quantity Surveying, Faculty of Built Environment, Universiti Malaysia Sarawak, Datuk Mohammad Musa Road, Kota Samarahan, 94300 Sarawak Malaysia; 4 Department of Paraclinical Science, Faculty of Medicine and Health Sciences, Universiti Malaysia Sarawak, Datuk Mohammad Musa Road, Kota Samarahan, 94300 Sarawak Malaysia

**Keywords:** Older adult, Psychosocial wellbeing, Questionnaire validation, Factor analysis, Malaysia

## Abstract

**Background:**

Malaysia’s ageing population highlights the need for robust instruments to assess psychosocial wellbeing among older adults, especially in semi-urban settings such as Sibu, Sarawak. However, many existing tools have limited cultural adaptation, unclear factor structure, or uncertain comparability across population subgroups. This study evaluated the internal consistency and factor structure of a 69-item psychosocial wellbeing questionnaire and conducted preliminary exploratory analyses of cross-group similarity by gender, recognizing that full measurement invariance testing was beyond the scope of this study.

**Methods:**

A community-based cross-sectional study was conducted among 1,077 community-dwelling older adults (≥ 60 years) in Sibu, Sarawak, selected using multistage stratified sampling. Interviewer-administered questionnaires captured sociodemographic characteristics, the 69-item psychosocial wellbeing instrument, and MHC–SF scores. Internal consistency was assessed using Cronbach’s α and McDonald’s ω. Factorability was evaluated with the Kaiser–Meyer–Olkin (KMO) index and Bartlett’s test of sphericity. Dimensionality was explored using parallel analysis and common factor analysis with principal axis-type extraction and varimax rotation. Preliminary cross-group stability across gender was examined using Tucker’s congruence coefficients.

**Results:**

The mean age of respondents was 67.1 years (SD 6.6); 52.0% were men and the largest ethnic group was Chinese (38.7%). Total scale reliability was excellent (α = 0.97; ω ≈ 0.97), while most subscales showed good internal consistency (α = 0.79–0.94). Block A demonstrated weaker reliability (α ≈ 0.39), indicating a need for item refinement. Factorability indices were strong (KMO ≈ 0.949; Bartlett’s χ² ≈ 72,586, *p* < 0.001). Parallel analysis supported retention of nine factors. Common factor analysis yielded a rotated solution that largely aligned with the intended content domains, although some overlap between blocks suggested conceptual proximity and possible redundancy. Tucker’s congruence analysis indicated strong cross-gender similarity for most factors (φ ≈ 0.89–0.99), with two factors showing only moderate similarity (φ ≈ 0.53–0.67).

**Conclusions:**

The 69-items instrument demonstrates excellent overall internal consistency and a coherent multi-factor structure among older adults in Sibu. At the same time, one subscale and a small number of factors show weaker performance and potential gender-related differences. These findings support cautious use of the instrument for research and programme evaluation in similar urban Sarawak settings, while underscoring the need for further refinement, full confirmatory factor analysis, measurement invariance testing and convergent validity assessment before widespread adoption.

**Supplementary Information:**

The online version contains supplementary material available at 10.1186/s12877-026-07257-5.

## Introduction

Malaysia, like many countries in the Asia Pacific region, is experiencing a rapid ageing transition, with the number of older adults increasing steadily. As people live longer, attention has shifted beyond disease and disability toward psychosocial wellbeing, which influences quality of life, independence, resilience, and social participation in later life. Psychosocial wellbeing is, however, a multidimensional concept shaped not only by emotional and psychological states, but also by social relationships, family support, community connectedness, and the physical and social environments in which older people live.

The World Health Organization (WHO) Age-Friendly Cities and Communities framework highlights how neighbourhood accessibility, social inclusion, safety, transportation, and community services influence wellbeing in older age. Complementing this, the Mental Health Continuum–Short Form (MHC–SF) conceptualises wellbeing across emotional, psychological, and social domains. Considered together, these frameworks suggest that psychosocial wellbeing arises from both internal experiences and external environmental supports. However, most available wellbeing instruments were developed in Western populations, and there remains a lack of culturally grounded tools that reflect the lived realities of older adults in Malaysia, particularly in East Malaysia and Sarawak, where social structures, intergenerational life, and community settings may differ.

To address this gap, our research team developed a 69-item psychosocial wellbeing questionnaire for community-dwelling older adults in Sarawak, drawing on the WHO Age-Friendly framework for environmental and social aspects and the MHC–SF for emotional and psychological elements.

Therefore, this study aimed to evaluate the reliability, factor structure, and preliminary cross-group stability of the 69-item psychosocial wellbeing questionnaire among older adults in Sibu, Sarawak. By providing local psychometric evidence, this study contributes to the growing field of ageing research in Malaysia and offers a culturally informed tool to support future work in community health, gerontology, and policy planning, while recognising that further refinement and confirmatory validation remain important next steps.

## Methods

Sibu was selected as the study site because it represents a typical urban center in East Malaysia with a rapidly growing older adult population, diverse ethnic composition, and established community infrastructure. As one of the major towns in Sarawak, Sibu provides an appropriate setting to examine psychosocial wellbeing within an urban context where older adults experience both age-friendly services and urban-related challenges. Studying Sibu allows insights into ageing in an East Malaysian urban environment, while recognizing that findings may not be directly generalizable to rural or institutionalized older populations. The inclusion criteria were the ability to communicate in Malay, English, or a local dialect, the absence of severe cognitive impairment, and consent to participate. Individuals who were bedridden, institutionalized, or diagnosed with dementia were excluded. To achieve a robust and representative sample, a total size of 1077 participants was calculated using a single population proportion formula. This estimation was based on a 95% confidence level and a 5% margin of error, incorporating a projected prevalence rate of 50% for individuals experiencing poor psychosocial well-being. Furthermore, an additional 20% was added to the final sample size to account for potential non-responses, and ensure the reliability of the findings.

A multistage stratified sampling method was employed throughout the southern zones of Sarawak. Initially, administrative districts in each city were stratified by population density. Subsequently, residential areas within selected divisions were randomly chosen. Finally, eligible older adults from households within these areas were selected using simple random sampling. Proportional allocation ensured representation from each city. Data was collected via in-person interviews using a standardized questionnaire administered by trained field enumerators.

The study was organized into three main parts. The initial part gathered data on socio-demographic characteristics of the respondents. The second part assessed psychosocial well-being utilizing the Mental Health Continuum, Short Form (MHC–SF). The assessment comprises 14 items that evaluate three areas: emotional well-being (composed of positive emotions and satisfaction with life), psychological well-being (comprising autonomy, personal growth, and self-acceptance), and social well-being (involving social integration, contribution, and coherence). Participants reported the frequency of these feelings they had experienced in the last month, utilizing Likert scale that ranged from “never” to “every day. The second part of the questionnaire assessed psychosocial well-being using the Mental Health Continuum–Short Form (MHC–SF), an instrument comprising 14 items that assessed emotional, psychological, and social well-being. Participants rated the frequency of specific feelings during the past month on a Likert scale ranging from (“never”) to (“every day”). According to established scoring methods, participants were classified with poor well-being (low scores in emotional and psychological or social items), moderate well-being, and good well-being (frequent experiences of positive states across all domains).

Data entry and cleaning were conducted using Microsoft Excel, and analysis done using the Statistical Package for the Social Sciences software version 28.0 (SPSS, IBM Corporation, Armonk, NY, USA). Descriptive statistics summarized the characteristics of the sample, which included means, standard deviations, frequencies, and percentages. The relationship between categories of psychosocial well-being (poor, moderate, good) and independent variables was evaluated using chi-square tests for categorical data. Variables displaying p-values of less than 0.20 in bivariate analysis were incorporated into an ordinal logistic regression model to calculate adjusted odds ratios (OR) and 95% confidence intervals (CI) for factors influencing psychosocial well-being. The criterion for statistical significance was set at *p* < 0.05. All statistical evaluations were carried out under the guidance of a senior biostatistician to ensure robustness and reproducibility.

### Instrument development

This study was conducted over 18 months, from April 2023 to October 2024, utilized a community-based cross-sectional design to investigate the sociodemographic factors affecting the psychosocial well-being of older adults, in urban areas of Sarawak: Sibu. The 69-item psychosocial wellbeing questionnaire was developed by the research team based on the WHO Age-Friendly Cities and Communities framework and the Mental Health Continuum–Short Form (MHC–SF). The WHO framework informed the environmental and social participation domains (Blocks B–H), including perceptions of neighborhood safety, transportation, housing, social support, and community engagement. The MHC–SF provided the conceptual basis for emotional, psychological, and social wellbeing, which guided item wording and response options for the core psychosocial domains (Blocks A–F). Together, these frameworks were used to operationalize a multidimensional construct of psychosocial wellbeing that captures both individual experiences and age-friendly contextual conditions.

Initial item generation involved iterative discussions among public health specialists, gerontologists, and clinical psychologists, followed by content review by a panel of five experts to assess relevance, clarity, and cultural appropriateness. A small pre-test among 30 older adults from a non-study area was conducted to refine wording and ensure comprehensibility. No formal pilot factor analysis was conducted prior to the present study; instead, this community survey represents the first full psychometric evaluation of the instrument in a large sample of older adults in Sarawak. It commenced after approval had been received from the Institutional Review Board.

### Scoring

Each item was rated on a 5-point Likert scale, with higher scores generally indicating better perceived psychosocial wellbeing or more favorable age-friendly conditions. For the current analysis, all items were scored in the same direction based on the original codebook; however, we acknowledge that some items in Block A are conceptually negative and may require reverse-scoring in future work. Subscale scores were computed as the sum of items within each block (A–H), and a total psychosocial wellbeing score was calculated by summing all 69 items.

### Reliability and factorability

Internal consistency of the total scale and each subscale was assessed using Cronbach’s α and McDonald’s ω [1]. Item–total correlations were examined to identify items contributing weakly to the total scale. Factorability was evaluated using the Kaiser–Meyer–Olkin (KMO) measure and Bartlett’s test of sphericity. Values of KMO ≥ 0.80 and a significant Bartlett’s test were considered indicative of suitability for factor analysis [2].

### Exploratory factor analysis and parallel analysis

In line with international psychometric guidelines (COSMIN), we treated the 69 items as indicators of latent constructs and therefore used a common factor approach rather than principal components analysis. A principal axis-type extraction method was applied to the item correlation matrix, followed by varimax rotation to aid interpretability. The number of factors to retain was guided by a combination of parallel analysis (Table 1), eigenvalues, scree plot inspection, and substantive interpretability. Parallel analysis was conducted using 100 randomly generated datasets with the same number of variables and sample size. Factors were retained when their observed eigenvalues exceeded the corresponding mean eigenvalues from random data.

**Table 1:** Summary of factor retention (parallel analysis)

Factor	Observed eigenvalue	Random eigenvalue
1	24.07	1.53
2	6.87	1.49
3	4.65	1.46
4	3.04	1.44
5	2.70	1.41
6	2.18	1.39
7	1.58	1.37
8	1.51	1.35
9	1.34	1.33
10	1.27	1.31

Although psychosocial constructs are theoretically correlated, varimax rotation was selected to enhance interpretability and parsimony in this initial exploratory evaluation. We acknowledge that oblique rotation may yield a more theoretically nuanced solution and recommend its use in future confirmatory analyses. This study represents the first large-scale psychometric evaluation of the 69-item psychosocial wellbeing questionnaire among older adults in Sarawak. Analyses focused primarily on internal consistency and exploratory assessment of dimensional structure. Cross-group analyses by gender were conducted for exploratory purposes only to provide an initial indication of structural similarity, and were not intended as formal tests of measurement invariance.

### Preliminary cross-group stability

To obtain an initial indication of whether the factor structure was similar across gender groups, we repeated the exploratory factor analysis separately among men and women and calculated Tucker’s congruence coefficients for the best-matched factors. Following common benchmarks, coefficients ≥ 0.90 were interpreted as strong similarity, 0.85–0.89 as fair, and < 0.85 as indicating that the factor structure might differ meaningfully between groups [3]. We emphasise that this approach provides only preliminary evidence. The full measurement invariance requires multi-group confirmatory factor analysis [4].

### Confirmatory factor analysis and measurement invariance

In next step, we specify a theory-driven CFA model in a structural equation modelling framework [5] and evaluate model fit using standard indices [6], followed by stepwise tests of configural, metric and scalar invariance across gender and other key subgroups.

## Results

A total of 1077 older adults participated in the study. The mean age was 67.1 years (SD = 6.6). Slightly more than half (52.0%) were male, and the majority (38.7%) were of Chinese ethnicity. Most participants were married (73.4%), had secondary school education or higher (62.0%), and reported a monthly household income below RM3000 (56.5%) (Table 2). Approximately 65.1% lived in households with fewer than five people. In terms of psychosocial well-being, 37.2% of respondents were classified as having low well-being, 31.9% with moderate, and 30.9% with high (Table 3). The proportion of high well-being was greater among younger participants (60–64 years) and those living in smaller households. Prevalence differences by gender, income, education, and marital status were not statistically significant in bivariate analysis.

**Table 2:** Sociodemographic characteristics of participants (*n* = 1077)

Variable	*n* (%)
Age (mean ± SD)	67.1 ± 6.6
Gender	
Male	560 (52.0)
Female	517 (48.0)
Ethnicity	
Chinese	417 (38.7)
Iban	198 (18.4)
Malay	178 (16.5)
Bidayuh	141 (13.1)
Melanau	98 (9.1)
Others	45 (4.2%)
Marital Status	
Married	791 (73.4)
Unmarried	286 (26.6)
Education level	
Secondary and above	667 (62.0)
Primary	312 (28.9)
None	98 (9.1)
Household Income	
<3000.00	608 (56.5)
>3000.00	469 (43.5)
Household Size	
<5	701 (65.1)
≥5	376 (34.9)

**Table 3:** Distribution of psychosocial well-being levels

Category	*n* (%)
Poor	400 (37.2)
Moderate	343 (31.9)
Good	334 (30.9)

### Reliability

Subscale scores demonstrated good to excellent internal consistency, with Cronbach’s α ranging from 0.79 to 0.94 for Blocks B–H (Table 4). Block A demonstrated lower but acceptable internal consistency (Cronbach’s α = 0.678) compared with other subscales. This suggests possible conceptual heterogeneity or the influence of negatively worded items that were not reverse-scored, rather than a complete lack of reliability. The total 69-item scale showed excellent internal consistency (α ≈ 0.97), and McDonald’s ω for the overall score was also high (ω ≈ 0.97), supporting the reliability of the composite psychosocial wellbeing index.

**Table 4:** Internal consistency of questionnaire subscales (Cronbach’s α)

Subscale	No. of items	Cronbach’s α
Block A	5	0.678
Block B	10	0.933
Block C	8	0.788
Block D	5	0.868
Block E	5	0.900
Block F	9	0.936
Block G	8	0.932
Block H	8	0.952
KP domain	11	0.919
Total 69-item scale	69	0.970

### Factorability and dimensionality

The Kaiser–Meyer–Olkin measure was 0.949, indicating excellent sampling adequacy. Bartlett’s test of sphericity was significant (χ² = 72,586; df = 2,415; *p* < 0.001), confirming the suitability of the data for factor analysis (Table 5). Parallel analysis suggested that nine factors should be retained, as the first nine observed eigenvalues exceeded those from randomly generated datasets. The rotated common factor solution accounted for a substantial proportion of variance in item responses and yielded interpretable clusters that largely corresponded to the conceptual blocks, such as neighbourhood and built environment (H), social relationships, and support (B, G), and functional or role-related aspects (F).

**Table 5:** Sampling adequacy and sphericity tests

Test	Statistic	df	*p*-value	Interpretation
Kaiser–Meyer–Olkin (KMO)	0.949	–	–	Sampling adequacy
Bartlett’s test of sphericity	72,586	2415	**< 0.001**	Correlations suitable for factor analysis

*p* < 0.05 indicates statistical significance

Some factors combined items from different blocks, reflecting conceptual overlaps (e.g. between safety and social participation), and several items in Block A loaded weakly or cross-loaded, consistent with its lower reliability. Full item-level factor loadings and cross-loadings are provided in the Supplementary Tables 1, to allow detailed evaluation of individual item performance.

### Preliminary cross-group stability

Tucker’s congruence coefficients were used as an exploratory assessment of cross-gender similarity in factor structure and should be interpreted as preliminary, as formal measurement invariance testing was not conducted. Tucker’s congruence coefficients for the best-matched factors across men and women indicated strong similarity (φ ≈ 0.89–0.99) for seven of the nine factors (Table 6). Two factors showed only moderate similarity (φ ≈ 0.53 and 0.67), driven mainly by items with weaker loadings and cross-loadings. These findings suggest that the core latent structure is broadly comparable across gender, but some dimensions may function differently for men and women and would benefit from item refinement and formal multi-group CFA before using the instrument for strict cross-group comparisons.

**Table 6:** Tucker’s congruence coefficients (gender-specific factor solutions)

Factor	φ (male vs. female)	Interpretation
F1	0.99	Strong similarity
F2	0.97	Strong similarity
F3	0.94	Strong similarity
F4	0.93	Strong similarity
F5	0.92	Strong similarity
F6	0.89	Fair similarity
F7	0.88	Fair similarity
F8	0.67	Moderate similarity
F9	0.53	Moderate similarity

Table 7 shows the results of the proportional-odds ordinal logistic regression. After adjusting for other variables, none of the sociodemographic characteristics examined were significantly associated with higher psychosocial wellbeing, as all 95% confidence intervals crossed unity. The ordinal logistic regression analysis was conducted as a secondary, exploratory analysis to describe associations between psychosocial wellbeing categories and sociodemographic variables, and was not intended as a primary psychometric objective.

**Table 7:** Ordinal logistic regression of factors associated with psychosocial wellbeing

Variable	Adjusted OR	95% CI
Male (vs. Female)	1.10	0.88–1.37
Age 65–69 (vs. 60–64 years)	1.05	0.80–1.38
Age ≥ 70 (vs. 60–64 years)	0.86	0.66–1.12
Secondary and above education (vs. Primary and below)	1.20	0.73–1.96
Household income ≥ RM3000 (vs. < RM3000)	1.01	0.77–1.33
Household size ≥ 5 (vs. < 5 members)	1.10	0.88–1.38

## Discussion

Our findings show that the 69-item instrument has excellent overall internal consistency and a coherent multi-factor structure among older adults in Sibu, consistent with its joint grounding in the WHO Age-Friendly Cities and Communities framework and the MHC–SF. Most subscales performed well psychometrically, with high reliability and clear factor loadings, suggesting that older people in this multi-ethnic urban Sarawak setting respond in a systematic way to items capturing neighbourhood environment, social support and participation, and perceived functional roles.

At the same time, the weaker reliability for Block A and the moderate cross-gender congruence observed for two factors highlight important areas for refinement. Items in Block A may be conceptually diverse and include negatively worded statements that were not reverse-scored in the present analysis, which can reduce internal consistency and distort factor loadings. Future work should revisit the wording, response direction, and content coverage of these items, potentially removing or rewriting poorly performing indicators and testing alternative factor models using confirmatory factor analysis.

The partial overlap between blocks in the rotated factor solution may also reflect the lived reality of ageing in Sibu, where psychosocial wellbeing is shaped by intertwined experiences of physical environment, social relationships, and economic security. In such contexts, clear statistical separation between domains may be difficult to achieve, and some redundancy between items is expected. Rather than viewing this as a weakness, we interpret it as an opportunity to streamline the instrument by focusing on items that both loads strongly on their target factor and remain robust across gender and other key subgroups.

In our setting, psychosocial wellbeing did not differ significantly by basic sociodemographic characteristics. This suggests that wellbeing among older people in Sibu may be shaped more strongly by personal, relational, and environmental experiences than by demographic position alone. However, larger studies in other Malaysian populations will be useful to verify this pattern.

Taken together, these psychometric results suggest that the instrument is suitable for use in community surveys and programme evaluations among older adults in similar urban Sarawak settings, provided the findings especially subgroup comparisons, are interpreted with caution. Before large-scale adoption, formal CFA, measurement invariance testing and convergent and discriminant validity assessments with external criteria are needed to consolidate its status as a fully validated measure. Although preliminary analyses suggested broadly similar factor structures across gender, these findings should be interpreted with caution. The gender comparisons in this study were exploratory in nature and do not constitute formal measurement invariance testing [7]. Full multi-group confirmatory factor analysis is required before the instrument can be used confidently for direct gender comparisons.

## Conclusions

The instrument demonstrates robust reliability and coherent structure for elderly in Sibu. Future work should confirmatory test measurement invariance across subgroups using CFA and evaluate convergent validity [8].

## Limitations

This study has several limitations. First, although we used a common factor approach for the present analysis, the instrument was originally analysed using principal components analysis, which is not optimal for modelling latent constructs [9]. Our current factor analytic results should therefore be considered exploratory and require confirmation in an independent dataset using full structural equation modelling. Second, we did not conduct a formal confirmatory factor analysis or report model fit indices such as CFI, TLI, RMSEA and SRMR [10]. These will be important next steps in future research to test theory-driven models and evaluate measurement invariance across gender, age and ethnic groups. Third, the present study did not examine convergent and discriminant validity with external measures of mental health or quality of life, nor did it apply item response theory models, which could provide deeper insights into item functioning and differential item performance.

Fourth, the instrument was tested only in one urban district in Sarawak, and participants were community dwelling older adults who were able to participate in face-to-face interviews. The findings may therefore not be generalisable to older people in rural areas, institutional settings, or other Malaysian states with different socio-cultural profiles. Finally, some items, particularly in Block A may require reverse-scoring or conceptual refinement, and our current scoring approach may underestimate the reliability and structural clarity of this domain.

Exclusion of older adults with dementia or severe cognitive impairment may limit the applicability of the instrument to healthier, community-dwelling populations, and further validation is required before use in cognitively impaired or institutionalized groups.

## Supplementary Information

Below is the link to the electronic supplementary material.

Supplementary Material 1.

Supplementary Material 2.

## Data Availability

The dataset analyzed during the current study is available from the corresponding author on reasonable request.
