# Supplementary material for: Psychometric evaluation of a psychosocial wellbeing questionnaire for older adults in Sibu, Sarawak, Malaysia
Source: BMC Geriatr. 2026 Mar 4;26:505. doi: 10.1186/s12877-026-07257-5 (PMC13067645; doi:10.1186/s12877-026-07257-5)
Supplement: Supplementary file 1 — Supplementary Material 1. [file 12877_2026_7257_MOESM1_ESM.pdf]

**Questionnaire**

**DATA COLLECTION DETAILS (for official use only and not to be analysed)**

Division : ...........................

District : ...........................

Name of city/village : ...........................

Household ID : ...........................

**Part 1: Demographic and socioeconomic characteristics**

I would like to ask a few questions about your demographic and socioeconomic characteristics. If you have any problem understanding the questions, please inform me. Please be truthful in your responses and **<u>CIRCLE ONLY ONE</u>** response appropriately in the code number given.

| No | Question | Response | |
|---|---|---|---|
| 1. | How old are you? | ...... years old | |
| 2. | What is your gender/sex? | Male = 1<br>Female = 2 | |
| 3. | What is your race group? | Malay = 1<br>Iban = 2<br>Chinese = 3<br>Bidayuh = 4<br>Melanau = 5<br>Others (Specify) = 6 | |
| 4. | What is your religion? | Islam = 1<br>Christianity = 2<br>Buddhism = 3<br>Others (Specify) = 4 | |
| 5. | What is your highest education level? | No formal education = 1<br>Primary school = 2<br>Secondary school (lower) = 3<br>Secondary school (upper) = 4<br>Pre-university = 5<br>Tertiary = 6<br>Others (Specify) = 7 | |
| 6. | What is your occupation? | No more working = 1<br>Working, please specify job types = 2 | |
| 7. | What is your marital status? | Married =1 | |

| No | Question | Response | |
|---|---|---|---|
| | | Not married =2<br>Divorced/widowed =3 | |
| 8. | How many family members stay with you in your house? | …… people | |
| 9. | What is your family member's monthly income? | RM……….. | |
| 10. | Do have any health problem? | No = 1<br>If Yes, please specify = 2 | |

**Part 2: The Age-Friendly Cities and Communities Questionnaire (AFCCQ)**

I would like to ask a few questions about your opinion of your neighbourhood environment.

If you have any problem understanding the questions, please inform me. Please **CIRCLE** only **ONE** most appropriate response.

**Section 1: Housing**

| No | Statement | 1 Strongly Disagree<br>10 Strongly Agree | | | | | | | | | |
|---|---|---|---|---|---|---|---|---|---|---|---|
| 1 | My house location is easily accessible to me. | 1 | 2 | 3 | 4 | 5 | 6 | 7 | 8 | 9 | 10 |
| 2 | My house is accessible to the people who come to visit me. | 1 | 2 | 3 | 4 | 5 | 6 | 7 | 8 | 9 | 10 |
| 3 | I feel burdened by the housing-related cost. | 1 | 2 | 3 | 4 | 5 | 6 | 7 | 8 | 9 | 10 |
| 4 | My current home is well equipped with basic utilities (e.g., water, electricity, and others). | 1 | 2 | 3 | 4 | 5 | 6 | 7 | 8 | 9 | 10 |
| 5 | I want to renovate my house to avoid accidents and physical inconvenience (grab bar, anti-slippery device, barrier-free device, etc.). | 1 | 2 | 3 | 4 | 5 | 6 | 7 | 8 | 9 | 10 |

**Section B: Social participation**

| No | Statement | 1 Strongly Disagree<br>10 Strongly Agree | | | | | | | | | |
|---|---|---|---|---|---|---|---|---|---|---|---|
| 1 | There are ample opportunities to meet people in my neighbourhood. | 1 | 2 | 3 | 4 | 5 | 6 | 7 | 8 | 9 | 10 |
| 2 | There are activities organised that are accessible to me. | 1 | 2 | 3 | 4 | 5 | 6 | 7 | 8 | 9 | 10 |
| 3 | Information about activities and events is suitable for me. | 1 | 2 | 3 | 4 | 5 | 6 | 7 | 8 | 9 | 10 |
| 4 | I find the events and activities are varied. | 1 | 2 | 3 | 4 | 5 | 6 | 7 | 8 | 9 | 10 |
| 5 | The venues for most of the events are easy to be visited. | 1 | 2 | 3 | 4 | 5 | 6 | 7 | 8 | 9 | 10 |
| 6 | Activities organized in neighbourhood | 1 | 2 | 3 | 4 | 5 | 6 | 7 | 8 | 9 | 10 |

| | | | | | | | | | | | |
|---|---|---|---|---|---|---|---|---|---|---|---|
| | are convenient to me. | | | | | | | | | | |
| 7 | Information about community events such as how to participate, how to use facilities, and transportation routes are easy to obtain. | 1 | 2 | 3 | 4 | 5 | 6 | 7 | 8 | 9 | 10 |
| 8 | There are many opportunities to participate in various social activities (religious, cultural gatherings, leisure activities, hobbies, and so on). | 1 | 2 | 3 | 4 | 5 | 6 | 7 | 8 | 9 | 10 |
| 9 | There are many opportunities for me to join volunteer services. | 1 | 2 | 3 | 4 | 5 | 6 | 7 | 8 | 9 | 10 |
| 10 | My local authority and my community provide ongoing assistance. | 1 | 2 | 3 | 4 | 5 | 6 | 7 | 8 | 9 | 10 |

**Section C: Respect and social inclusion**

| No | Statement | 1 Strongly Disagree<br>10 Strongly Agree | | | | | | | | | |
|---|---|---|---|---|---|---|---|---|---|---|---|
| 1 | Sometimes I get annoying or negative statements due to my age. | 1 | 2 | 3 | 4 | 5 | 6 | 7 | 8 | 9 | 10 |
| 2 | Sometimes I face discrimination because of my age. | 1 | 2 | 3 | 4 | 5 | 6 | 7 | 8 | 9 | 10 |
| 3 | The people in my neighborhood are courteous and respectful of older adults. | 1 | 2 | 3 | 4 | 5 | 6 | 7 | 8 | 9 | 10 |
| 4 | The staff at the district office, community centre are helpful. | 1 | 2 | 3 | 4 | 5 | 6 | 7 | 8 | 9 | 10 |
| 5 | Activities and events are exciting for all generations. | 1 | 2 | 3 | 4 | 5 | 6 | 7 | 8 | 9 | 10 |
| 6 | I feel respected in social interactions. | 1 | 2 | 3 | 4 | 5 | 6 | 7 | 8 | 9 | 10 |
| 7 | Older adults are consulted by public, voluntary, and commercial services on how to better serve them. | 1 | 2 | 3 | 4 | 5 | 6 | 7 | 8 | 9 | 10 |
| 8 | Older adults are portrayed positively in public media (e.g., televisyen, newspaper and radio programme). | 1 | 2 | 3 | 4 | 5 | 6 | 7 | 8 | 9 | 10 |

**Section D: Civic participation and employment**

| No. | Statement | 1 Strongly Disagree<br>10 Strongly Agree | | | | | | | | | |
|---|---|---|---|---|---|---|---|---|---|---|---|
| 1 | I have ample opportunity to interact with younger generations. | 1 | 2 | 3 | 4 | 5 | 6 | 7 | 8 | 9 | 10 |
| 2 | I feel valued as a member of this society. | 1 | 2 | 3 | 4 | 5 | 6 | 7 | 8 | 9 | 10 |
| 3 | I can join activities that involve both younger and older people. | 1 | 2 | 3 | 4 | 5 | 6 | 7 | 8 | 9 | 10 |
| 4 | There are variety of cultural activities for diverse populations. | 1 | 2 | 3 | 4 | 5 | 6 | 7 | 8 | 9 | 10 |
| 5 | Older adults have opportunities to participate in the committees. | 1 | 2 | 3 | 4 | 5 | 6 | 7 | 8 | 9 | 10 |

**Section E: Communication and information**

| No. . | Statement | 1 Strongly Disagree<br>10 Strongly Agree | | | | | | | | | |
|---|---|---|---|---|---|---|---|---|---|---|---|
| 1 | Printed and digital information is easy to read (in terms of their font and size). | 1 | 2 | 3 | 4 | 5 | 6 | 7 | 8 | 9 | 10 |
| 2 | Printed and digital information are written in simple language. | 1 | 2 | 3 | 4 | 5 | 6 | 7 | 8 | 9 | 10 |
| 3 | There is free access to computers and the Internet in public places. | 1 | 2 | 3 | 4 | 5 | 6 | 7 | 8 | 9 | 10 |
| 4 | Community information are delivered to people at their home. | 1 | 2 | 3 | 4 | 5 | 6 | 7 | 8 | 9 | 10 |
| 5 | There is access to community information in one central source. | 1 | 2 | 3 | 4 | 5 | 6 | 7 | 8 | 9 | 10 |

**Section F: Community support and health services**

| No. | Statement | 1 Strongly Disagree<br>10 Strongly Agree | | | | | | | | | |
|---|---|---|---|---|---|---|---|---|---|---|---|
| 1 | The supply of care and welfare in my city is adequate for me. | 1 | 2 | 3 | 4 | 5 | 6 | 7 | 8 | 9 | 10 |
| 2 | When I am sick, I receive the care and help I need. | 1 | 2 | 3 | 4 | 5 | 6 | 7 | 8 | 9 | 10 |
| 3 | I can contact care and welfare services by telephone and in person. | 1 | 2 | 3 | 4 | 5 | 6 | 7 | 8 | 9 | 10 |
| 4 | I have information about care and welfare services in my neighbourhood. | 1 | 2 | 3 | 4 | 5 | 6 | 7 | 8 | 9 | 10 |
| 5 | Care and welfare workers in my neighbourhood are sufficiently respectful. | 1 | 2 | 3 | 4 | 5 | 6 | 7 | 8 | 9 | 10 |
| 6 | The health centre and the welfare centres are easy and safe for me. | 1 | 2 | 3 | 4 | 5 | 6 | 7 | 8 | 9 | 10 |
| 7 | The staff at public facilities (e.g., hospitals) are helpful when I make inquiries. | 1 | 2 | 3 | 4 | 5 | 6 | 7 | 8 | 9 | 10 |
| 8 | I have easy access to programmes and information on health education, nutrition class, physical therapy and others. | 1 | 2 | 3 | 4 | 5 | 6 | 7 | 8 | 9 | 10 |
| 9 | In public facilities I can use the Internet for free or for a small fee. | 1 | 2 | 3 | 4 | 5 | 6 | 7 | 8 | 9 | 10 |

**Section G: Outdoor spaces and building**

| No. | Statement | 1 Strongly Disagree<br>10 Strongly Agree | | | | | | | | | |
|---|---|---|---|---|---|---|---|---|---|---|---|
| 1 | My neighbourhood are accessible by wheelchair users or wheeled walker users. | 1 | 2 | 3 | 4 | 5 | 6 | 7 | 8 | 9 | 10 |
| 2 | The shops in my neighbourhood are accessible by wheelchair users or wheeled walker users. | 1 | 2 | 3 | 4 | 5 | 6 | 7 | 8 | 9 | 10 |
| 3 | The public spaces in my neighborhood are clean and well maintained (e.g., (walking trails, parks, roads, sidewalks) | 1 | 2 | 3 | 4 | 5 | 6 | 7 | 8 | 9 | 10 |
| 4 | Walkaways and parks are easily accessible from | 1 | 2 | 3 | 4 | 5 | 6 | 7 | 8 | 9 | 10 |

| | | | | | | | | | | | |
|---|---|---|---|---|---|---|---|---|---|---|---|
| | my home. | | | | | | | | | | |
| 5 | The sidewalk area has a flat surface and is free of obstacles. | 1 | 2 | 3 | 4 | 5 | 6 | 7 | 8 | 9 | 10 |
| 6 | The traffic signals give me enough time to cross the roads safely. | 1 | 2 | 3 | 4 | 5 | 6 | 7 | 8 | 9 | 10 |
| 7 | At intersections, drivers give way to pedestrians to cross safely. | 1 | 2 | 3 | 4 | 5 | 6 | 7 | 8 | 9 | 10 |
| 8 | Cyclists are considerate and give way to pedestrians. | 1 | 2 | 3 | 4 | 5 | 6 | 7 | 8 | 9 | 10 |

**Section H: Transportation**

| No. | Statement | 1 Strong Disagree<br>10 Strongly Agree | | | | | | | | | |
|---|---|---|---|---|---|---|---|---|---|---|---|
| 1 | The bus and taxi stops in my neighbourhood are easy to reach and use. | 1 | 2 | 3 | 4 | 5 | 6 | 7 | 8 | 9 | 10 |
| 2 | I can easily get on the bus or taxi in my neighbourhood. | 1 | 2 | 3 | 4 | 5 | 6 | 7 | 8 | 9 | 10 |
| 3 | Public transportation is reliable and frequent. | 1 | 2 | 3 | 4 | 5 | 6 | 7 | 8 | 9 | 10 |
| 4 | Public vehicles are accessible and offer priority seating for the older adults. | 1 | 2 | 3 | 4 | 5 | 6 | 7 | 8 | 9 | 10 |
| 5 | Special transportation services are available for people with disabilities and older adults. | 1 | 2 | 3 | 4 | 5 | 6 | 7 | 8 | 9 | 10 |
| 6 | Bus drivers are careful and considerate when loading and unloading passengers to ensure their safety. | 1 | 2 | 3 | 4 | 5 | 6 | 7 | 8 | 9 | 10 |
| 6 | It is easy for me to use buses or taxis to get to where I want to go. | 1 | 2 | 3 | 4 | 5 | 6 | 7 | 8 | 9 | 10 |
| 8 | Bus stops have shaded benches to provide comfort. | 1 | 2 | 3 | 4 | 5 | 6 | 7 | 8 | 9 | 10 |

**Part C: Psychosocial wellbeing**

Please answer the following questions are about how you have been feeling during the past month. Please **CHOOSE** only **ONE** most appropriate response that best represents how you have experienced or felt the following:

| No. | During the past month, you feel | 1 Strong Disagree<br>10 Strongly Agree | | | | | | | | | |
|---|---|---|---|---|---|---|---|---|---|---|---|
| 1 | you had something important to contribute to society. | 1 | 2 | 3 | 4 | 5 | 6 | 7 | 8 | 9 | 10 |
| 2 | you belonged to a community (like a social group, or your neighborhood). | 1 | 2 | 3 | 4 | 5 | 6 | 7 | 8 | 9 | 10 |
| 3 | our society is a good place, or is becoming a better place, for everyone. | 1 | 2 | 3 | 4 | 5 | 6 | 7 | 8 | 9 | 10 |
| 4 | people around me are basically good. | 1 | 2 | 3 | 4 | 5 | 6 | 7 | 8 | 9 | 10 |
| 5 | the way our society works makes sense to you. | 1 | 2 | 3 | 4 | 5 | 6 | 7 | 8 | 9 | 10 |
| 6 | you liked most of your personality. | 1 | 2 | 3 | 4 | 5 | 6 | 7 | 8 | 9 | 10 |
| 7 | good at managing the responsibilities of your daily life. | 1 | 2 | 3 | 4 | 5 | 6 | 7 | 8 | 9 | 10 |
| 8 | you had warm and trusting relationships with others. | 1 | 2 | 3 | 4 | 5 | 6 | 7 | 8 | 9 | 10 |
| 9 | you had experiences that challenged you to grow and become a better person. | 1 | 2 | 3 | 4 | 5 | 6 | 7 | 8 | 9 | 10 |
| 10 | confident to think or express your own ideas and opinions. | 1 | 2 | 3 | 4 | 5 | 6 | 7 | 8 | 9 | 10 |
| 11 | your life has a sense of direction or meaning to it. | 1 | 2 | 3 | 4 | 5 | 6 | 7 | 8 | 9 | 10 |
