# Supplementary material for: Psychometric evaluation of a psychosocial wellbeing questionnaire for older adults in Sibu, Sarawak, Malaysia
Source: BMC Geriatr. 2026 Mar 4;26:505. doi: 10.1186/s12877-026-07257-5 (PMC13067645; doi:10.1186/s12877-026-07257-5)
Supplement: Supplementary file 2 — Supplementary Material 2. [file 12877_2026_7257_MOESM2_ESM.docx]

# Supplementary Table S1. Rotated factor loadings and cross-loadings for the 69-item psychosocial wellbeing questionnaire

Loadings are based on common factor analysis using principal axis factoring with varimax rotation. Only loadings ≥ 0.30 are shown. Primary loadings are shown in bold.

| **Item** | **Block** | **F1** | **F2** | **F3** | **F4** | **F5** | **F6** | **F7** | **F8** | **F9** |
| --- | --- | --- | --- | --- | --- | --- | --- | --- | --- | --- |
| **H4** | **H** | 0.845 |  |  |  |  |  |  |  |  |
| **H3** | **H** | 0.836 |  |  |  |  |  |  |  |  |
| **H7** | **H** | 0.830 |  |  |  |  |  |  |  |  |
| **H2** | **H** | 0.827 |  |  |  |  |  |  |  |  |
| **H1** | **H** | 0.817 |  |  |  |  |  |  |  |  |
| **H6** | **H** | 0.718 |  |  |  |  |  |  |  |  |
| **H8** | **H** | 0.701 |  |  |  |  |  |  |  |  |
| **H5** | **H** | 0.623 |  |  |  |  | 0.448 |  |  |  |
| **G7** | **G** | 0.501 | 0.368 |  | 0.493 |  |  |  |  |  |
| **G8** | **G** | 0.481 | 0.366 |  | 0.461 |  |  |  |  |  |
| **KP2** | **KP** | 0.448 |  |  |  |  |  |  |  |  |
| **KP1** | **KP** | 0.419 |  |  |  |  |  |  |  |  |
| **F2** | **F** |  | 0.820 |  |  |  |  |  |  |  |
| **F1** | **F** |  | 0.779 |  |  |  |  |  |  |  |
| **F3** | **F** |  | 0.763 |  |  |  |  |  |  |  |
| **F7** | **F** |  | 0.756 |  |  |  |  |  |  |  |
| **F4** | **F** |  | 0.742 |  |  |  |  |  |  |  |
| **F6** | **F** |  | 0.742 |  |  | 0.405 |  |  |  |  |
| **F5** | **F** |  | 0.700 |  |  | 0.446 |  |  |  |  |
| **F8** | **F** |  | 0.663 |  |  |  |  |  |  |  |
| **C4** | **C** |  |  | 0.596 |  |  | 0.315 |  |  |  |
| **C5** | **C** |  |  | 0.394 |  | 0.366 | 0.357 |  |  |  |
